# “DNA Doesn’t Lie:” Genetic Essentialism and Determinism in *Law & Order: Special Victims Unit*

**DOI:** 10.1007/s10912-024-09923-4

**Published:** 2025-01-24

**Authors:** Alice Lillydahl, Jay Clayton

**Affiliations:** Department of English, Vanderbilt University, Nashville, TN 37212 USA

**Keywords:** Police procedurals, CSI effect, *Law and Order: Special Victims Unit*, Genetic essentialism, Genetic determinism, Nature and nurture

## Abstract

*Law and Order: Special Victims Unit* (*SVU*) (1999–present) is a popular primetime drama that spotlights the use of genetic information to solve crimes. Despite the show’s heavy reliance on the forensic use of DNA evidence, the role of genetics in defining family and identity arises in complex ways. Many episodes wrestle with social, ethical, and legal questions that reflect assumptions about genetic essentialism and genetic determinism, but counterarguments about the importance of non-biological relationships, social factors, and legal entitlements are given equal or greater weight. For this study, we identified and viewed 38 episodes from *SVU*’s first twenty seasons centered on genetic themes in non-forensic contexts. Two recurring themes emerged: (1) that the role DNA plays is only one factor in a complex web of biological and social considerations that shape our understanding of kinship; and (2) that genetic predispositions to behavioral traits such as mental illness or violence should not be seen as obscuring the responsibility of personal choice. By treating genetics as a complex source of information requiring social context to be understood, *SVU* allows audiences to play an active role in interpreting its meaning.

## Introduction

Forensic crime dramas, a descendant of the television genre known as “police procedurals,” are immensely popular in the USA (Koblin 2023). Television police procedurals date back to the 1950s, but forensic crime dramas grew to prominence in the 1970s, with shows such as *Quincy M.E.* (NBC, 1976–83) drawing large audiences. The genre expanded again in the twenty-first century, when *Law and Order: Special Victims Unit* (*SVU*) (NBC, 1999–present) and *CSI: Crime Scene Investigation* (2000–present) inspired numerous imitators such as *Bones* (Fox, 2005–present), *NCIS* (CBS, 2003–present), and two *CSI* spinoffs, *CSI: Miami* (CBS, 2005–10) and *CSI: NY* (CBS, 2004–13) (Bull 2015; Kirby 2013). One prominent tool used by detectives in forensic crime dramas is genetic evidence. Studies by Gayle Rhineberger-Dunn and her colleagues found that DNA was mentioned in 73.3% of sampled episodes of crime dramas (Rhineberger-Dunn et al. 2017). *SVU* is no exception: DNA was collected in 50% of cases depicted in the series and used to identify an initial suspect in 22% of sampled episodes (Rhineberger-Dunn et al. 2016).

*SVU* is the longest-running and most popular police procedural in television history, attracting an average of 6.5 million viewers per episode (Berr 2020). Old episodes of the show remain in circulation, appearing multiple times a week on cable and streaming channels. The formulaic structure of *SVU* compresses a crime, investigation, and trial into each hour-long episode. Often glorifying policing, *SVU* showcases the lives of fictional detectives in the New York City Police Department who specialize in solving sex offenses. Given this focus, it is unsurprising that DNA evidence is often front and center. The storylines concentrate on the criminal proceedings, but the detectives contextualize the crime story with their emotional reactions, which often reflect ongoing issues in their personal lives. The mixture of forensic realism (Jermyn 2013) and melodrama have made this show a favorite with female viewers (Little 2015; Rosenberg 2015) and given Detective Benson’s character a reputation as a feminist icon.^1^

Researchers have demonstrated that police procedurals can influence the opinions of their viewers on topics such as plea bargaining (Johnson 2019), police–community relations (Levan and Stevenson 2019), rape (Hust et al. 2015), and male rejection of traditional gender roles (Lee et al. 2010). Much of the existing literature on forensic crime dramas centers on the so-called *CSI* effect, which posits that jury members exposed to police procedurals have a higher expectation of forensic evidence in trials—both that such evidence will be used in actual trials and that it is particularly probative (Hayes and Levett 2012; Ley et al. 2010; Rhineberger-Dunn et al. 2017; Robbers 2008). These assumptions create real-life consequences for defendants and the prosecution due to increased pressure on the prosecution to produce genetic evidence. Without forensic evidence, many jurors exposed to police procedurals are less likely to issue a conviction (Rhineberger-Dunn et al. 2017).

One limitation of existing studies of the *CSI* effect is that they focus primarily on genetic testing in the context of criminal forensics. In *SVU,* however, genetic messaging extends beyond policing into other aspects of its characters’ lives. There are many episodes in which DNA collected in the course of a criminal investigation impacts individuals and families in ways that are unrelated to the criminal investigation. For example, genetic samples sometimes reveal biological connections between characters who are complete strangers to one another.

Such revelations—on the show and in real life—can have far-reaching effects on people’s understanding of their identity and family relations. Nelson and Hertz (2018) detail instances in real life in which individuals value a newly discovered genetic relationship over and above existing non-biological ties, which raises the specter of genetic essentialism. Other episodes of the show involve characters who believe that DNA can shape a person’s current and future behavior, a belief characteristic of genetic determinism.

The concept of genetic essentialism posits that a person’s behavior, characteristics, and identity are derived from their genetic makeup (Dar-Nimrod and Heine 2011; Harden 2022; Tabb et al. 2018). To believe in genetic essentialism is to suppose that “genes can reveal to us the core of what it means to be humans and individuals, that our genes are the ultimate source of who we are” (Halverson 2022, 397). In the same vein, genetic determinism names the tendency to overestimate the role that genes play in influencing behavior. In popular culture, the idea of genetic determinism is encapsulated in the slogan, “My genes made me do it” (Frentz et al. 2015). While genetic determinism might be seen as a subset of genetic essentialism, the two concepts can be distinguished for the purpose of analysis. The error of genetic essentialism is thinking that your genes constitute who you are; the error of genetic determinism involves thinking your genes dictate what you will do. Both concepts are displayed, for example, when the protagonist of the show, Detective Benson, runs her DNA through a law enforcement database and discovers she has a half-brother, Simon Marsden (Michael Weston) (S8.E16). Benson believes Simon may be guilty of stalking a woman, but he defends himself, telling her “Oh because my dad raped your mom, now I’m a rapist too?” Here, Simon rejects Benson’s genetic determinist assumption that he would commit crimes against women because he shares genetic material with a rapist. Later in the episode, Simon asks Benson about her taste in food and music. When their answers match, Simon says, “It’s in the blood,” implying the genetic essentialist view that they are the same because they are genetically related.

Commentators have sought for decades to debunk simplistic conceptions of both genetic essentialism and determinism (Dar-Nimrod and Heine 2011; Donovan 2022; Hubbard and Wald 1993; Keller 1995; Nelkin and Susan Lindee 1995). But in recent years, lawyers for defendants in criminal cases have argued that genetic predispositions for rape, violence, or mental illness should absolve their clients of guilt for their actions (Greely and Farahany 2019). As genetic technologies have changed over the lifespan of the show—and legal strategies have evolved in response—*SVU* has been quick to seize on both as sources for plotlines in their series.

Concern about the possible misunderstandings that might arise from storylines that exaggerate the importance of genetics in shaping one’s identity or dictating one’s behavior date back to the same foundational studies of the treatment of genetics in popular culture by Nelkin and Lindee, and Hubbard referenced above. Equal concern has been expressed about the persistent tendency to exaggerate the distinction between nature and nurture, which are now considered complexly interrelated (Tabery 2014; Wachs 1992). To explore these concerns, we focus on *SVU* because it pays attention to genetic plotlines outside of criminal forensics. Does the formulaic, fast-paced show find a place for nuance when discussing these topics? Or does it fall into the trap of simplification, endorsing genetic essentialism and genetic determinism?

The line “DNA doesn’t lie” is delivered by *SVU* detectives five times in our surveyed episodes (S5.E5, S6.E12, S13.E4, S14.7, S17.E8), but the DNA evidence ends up leading investigators down the wrong path in three of those instances. The forensic evidence was correct, but other social factors led to a misinterpretation of the facts. It is this tension between a strict reliance on the results of forensic science and the complications that arise from social context that prompts our three research questions: (1) When, where, and how are genetic essentialism and determinism deployed in *SVU*? (2) Does the distinction between the forensic context, on the one hand, and the social context, on the other, create a false dichotomy between these spheres? and (3) In representing these spheres as distinct, does *SVU*, one of the most popular TV shows of all time, perpetuate a misunderstanding of the relationship between nature and nurture in the very moments it seems to undermine genetic essentialism and determinism?

“Background” and “Method” sections provide further background about *SVU* and discuss our method*.* The fourth section, “Defining the Family: Genetic Essentialism vs. Social Relations,” turns to episodes of the show that present contrasting visions of the role of biological relatedness in the characters’ understanding of family. These episodes often revolve around custody battles between parties who claim that genetic kinship outweighs the social bonds established by existing familial arrangements, an argument that relies on genetic essentialism. The fifth section, “Conflicting Attitudes Toward Genetic Determinism,” analyzes the show’s depiction of legal arguments over whether a supposed genetic predisposition to rape, violence, or mental illness should exonerate a criminal accused of a crime. The article concludes with a brief discussion of the implications of our findings for the study of television in bioethical research.

## Background

Most episodes of *Law and Order: SVU* follow a set formula. First, a crime occurs, and the detectives arrive at the scene. Once the investigation begins, the detectives use a standard set of police procedures such as interviewing witnesses, analyzing forensic evidence, and searching publicly available databases for information about suspects. The middle of the episode is less formulaic because, in most episodes, this period is where a complication is introduced. For example, a key witness might be murdered, important evidence might go missing, or the prime suspect might be cleared of wrongdoing. Once the detectives charge a suspect, the focus of the show shifts to the lawyers involved in the case. The episodes almost always end on a note of finality, reassuring viewers that the criminal will no longer be free to harm society.

In *SVU*, genetic information is portrayed as intimate and almost impossible to keep hidden. The detectives use diverse methods with varying levels of bioethical concerns to obtain genetic material. Often, the detectives will ask for a voluntary DNA sample. If refused, they will get a warrant or covertly take a DNA sample in legally permissible but sometimes devious ways. In one episode, the detectives set up a fake drunk driving checkpoint and used a breathalyzer to collect the suspect’s DNA (S17.E8). In some cases, a family member might unknowingly assist the investigation by showing up as a partial match in a genetic information database. Once the detectives obtain genetic material and identify a match, many revelations follow. Genetic information can uncover secrets such as incestuous relationships, extramarital affairs, unknown biological relations, and hidden paternity. Three parties must make meaning out of each piece of genetic information: the affected parties (victims, suspects, witnesses), the detectives, and the lawyers and judges. Each party has a different stake in the outcome of the case, which often places them in conflicting positions in relation to the genetic information collected during an investigation. The most stable, recurring characters are the detectives and the team of specialists who help them analyze the evidence of a crime. A second set of recurring characters are the Assistant District Attorneys (ADAs). This group becomes central to the plot when a case’s legal implications are not straightforward, or there is controversy in the courtroom. The ADAs have moments of caring and attachment to the victims but are primarily concerned with the bottom line of what will work in a trial. The third group of characters are the crime victims.^2^ These characters rotate weekly, rarely re-appearing in subsequent episodes. These rotating characters are unlikely to garner the same audience attachment as the detectives or ADAs. Each of the three parties interprets the genetic information presented in the show in their own way and according to their varying, often conflicting interests. Consequently, our analyses must be alert to the contending forces at play in the depiction of genetics.

Detective Olivia Benson (Mariska Hargitay) leads the team in navigating the contentious topics and difficult decisions that arise while investigating a crime.^3^ Storylines about Detective Benson’s private life constitute the most important examples of the show’s attention to genetics outside of criminal forensics. Detective Benson, who was conceived as a result of sexual assault, struggles with the possibility that she has inherited a genetic predisposition to violence.

Additionally, Benson’s father committed suicide—an act that raises even more questions in her mind about a possible hereditary predisposition toward mental illness. When Benson runs her DNA through a law enforcement database, she discovers that she also has a half-brother who is accused of a violent crime, reinforcing her worry about a possible predisposition to violence. To complicate matters even further, Benson adopts a child who is himself a child of rape, and Benson eventually ends up in a custody battle with the child’s biological grandmother.

## Method

To identify episodes of *SVU* that treat genetics in non-forensic contexts, we followed the method outlined in Gibbons et al. (2021). First, we collected plot summaries from Wikipedia of all 458 episodes aired in *SVU*’s first twenty seasons (1999–2019). As crowd- sourced summaries of the episodes, Wikipedia entries have an advantage over précis generated by the network or other commercial outlets in that Wikipedia entries capture features of the show that viewers found important enough to discuss. This approach “moves us one step closer to the public reaction to the film or episode rather than a single analyst’s perspective” (Gibbons et al. 2021, 6). We then examined each plot summary looking for references to key concepts: genetic conditions (e.g., “Noonan syndrome”), genetic kinship (e.g., “biological grandparent”), genetic material (e.g., “DNA sample”), genetic medical research, geneticists, pregnancy, paternity, heredity, and hereditary conditions (e.g., the “rape gene”), genetic evidence, and mentions of Benson’s complex familial connections, including any reference to her father, adopted child, or biological relatives of that child. Our examination turned up 56 episodes that mentioned one or more of these key concepts; of those episodes, 43 featured plotlines involving genetics in non-forensic contexts.

From this initial examination, we determined that two themes dominated discussions of genetics in non-forensic contexts: first, that the role DNA plays was only one factor in a complex web of biological and social considerations that shape our understanding of kinship (29 episodes), and second, that a hereditary predisposition to behavioral traits such as mental illness or violence should not be seen as obscuring the responsibility of personal choice (7 episodes).

The first of these themes can be seen as challenging the idea of genetic essentialism, and the second as challenging the idea of genetic determinism. Using these two themes as a basis of selection, we chose 38 episodes for viewing in their entirety (a list of the episodes viewed may be found in the Appendix). As a body, these episodes provided the ground for exploring the treatment of DNA in forensic science vs. the context of personal and social relations (research question 2) and, indirectly, raised questions about the distinction between nature and nurture (research question 3).

As a final step prior to analyzing the episodes, we watched all 38 episodes, coding passages of dialogue, plot elements, and developments in the lives of characters that reflected the key concepts listed above. Our coding was designed to identify places where narrative developments in an episode changed how genetic information would be interpreted by the viewer. For example, a plot twist might discredit a character who believed that DNA trumped all other factors in determining parenthood, or a determinist argument about the role of genetics in shaping behavior might be expressed by a lawyer for the losing side in a case and disputed by the principal characters with whom the audience identifies. This method of analyzing coded passages combined techniques of textual analysis associated with film and television studies (Butler 2012) with qualitative methods and thematic analysis (Braun and Clarke 2021).

## Defining the family: Genetic essentialism vs. social relations

### Paternity

With a focus on sex offenses, *SVU* tends to make paternity the focal point of many investigations. Once a woman is discovered to be pregnant, either willingly or as a consequence of rape, she carries genetic evidence that often can solve a crime (see S1.E17; S4.E8; S4.E24; S5.E5; S7.E14; S8.E5; S13.E3; S19.E6; S20.E20). Despite the heavy reliance on genetic information to solve crimes, the detectives do not always defer to a biological explanation of fatherhood. A prime example of a paternity search occurs in “Confrontation” (S8.E5) when a rapist unexpectedly returns to attack his victims once per month, a pattern that corresponds to the victim’s ovulation cycles. One victim, Megan Carlisle, becomes pregnant.

Against her husband’s wishes, she agrees to undergo chorionic villus sampling to determine the paternity of her baby, which then identifies her rapist. Mr. Carlisle is distraught when he hears the news: “As long as I didn’t know, we were going to be all right!” The rapist, on the other hand, is overjoyed that he has a child and wants to sue for parental rights. Fortunately, the rapist is sent to prison and is not allowed to claim the child. However, Mr. Carlisle finds himself unable to embrace the child. Detective Stabler urges the husband to accept the child as his own, but the husband’s belief that biology is what determines fatherhood prevents him from embracing the role of fatherhood. The husband’s position is a form of genetic essentialism because he allows genetic kinship to trump other potential forms of relationship. The detectives, on the other hand, endorse the importance of a social connection between parents and the child, rejecting the essentialist reasoning of both the biological father (i.e., the rapist) and the husband.

*SVU* also presents questions of paternity that arise after the parents have established socially based familial bonds and the child has developed a sense of identity within its perceived family structure (see S9.E9; S13.E3; S13.E20; S14.E7; S17.E8). In these cases, the show depicts the rejection of genetic essentialism as the moral high ground. In “Paternity” (S9.E9), a nanny is found raped and murdered in her employer’s home, which leads the detectives to collect genetic material from the employer, Jake Keegan, and his three-year-old son, Tommy Keegan. The nanny’s murder is easily solved, but the medical examiner who ran Jake and Tommy’s DNA discovers something unexpected—Jake is not Tommy’s biological father. Detectives Benson and Stabler argue over telling the parents, and Jake accidentally overhears the conversation. Despite the detective’s support, Jake is crushed.

Detective Stabler: “It doesn’t mean you love him any less.”

Jake Keegan: “What happens when he gets older and all he wants is his real father?”

Detective Stabler: “There are plenty of adopted kids that don’t care about their biological parents. Even the ones that do…”

Jake Keegan: “I didn’t adopt him. I watched my wife give birth to him. I cut the umbilical cord. He was mine.”

Detective Stabler: “He still is.”

When Tommy’s biological father files for custody, an enraged Jake kills his wife and attempts to commit suicide when he is apprehended by the detectives. Initially portrayed as a loving husband and stable father, Jake is transformed into a murderer who cannot imagine staying alive in this new reality. In centering genetic essentialism, Jake ruins his entire life and commits a horrible crime. In the end, the episode endorses the detective’s perspective that deeply felt social relationships should trump a purely biological connection. The child ends the episode in the hands of his biological father by default, leaving the child’s custody decided but the larger philosophical questions of fatherhood unanswered.

Social rather than genetic relationships are given precedence in several legal conflicts where the detectives are successful in defending against arguments in favor of a purely genetic bond. In “Taboo” (S7.E14), a college student abandons her newborn baby on the street, attempting to conceal that the child resulted from an incestuous relationship with her recently discovered biological father. When the college-aged mother is hospitalized for postpartum depression, Everett Drake—the baby’s father—attempts to gain custody.

Everett Drake: “He’s my son. I’m taking custody.”

Detective Benson: “That’s never gonna happen.”

Everett Drake: “We were just finishing up the paperwork.”

A.D.A. Casey Novak: “We just came to serve you with an injunction barring Mr. Drake from having any contact with the infant.”

Everett Drake: “I can give him a home. And love. He belongs with his father.”

A.D.A. Casey Novak: “But not with his grandfather.”

Mr. Drake offers benefits to the child in the form of “a home” and “love,” but his choice to knowingly engage in an incestuous relationship with his daughter denies him any credibility and negates his claim to the child, at least according to this show’s moral compass. The detectives and the A.D.A. were able to convince a judge that the incestuous origin of the father-child relationship overrides genetic kinship.

In a few episodes, sons and daughters learn the truth of their parentage and grapple with the changed landscape of their relationship with the man they had long regarded as their father in light of their new knowledge about their biological kinship. In “Father Dearest” (S13.E20), teenage Cate learns that her mother secretly went to a sperm bank to conceive her; seeking love and reassurance, Cate allows a man pretending to be her biological father to seduce her. In “Blood Brothers” (S13.E3), thirteen-year-old Arturo is distraught when he discovers that his biological father is a wealthy, powerful man, and Arturo kills his privileged half-brother, who sexually assaulted Arturo’s best friend. Confronting his father, Arturo cries, “I just wanted you to call me your son. I just wanted you to say you love me. Why couldn’t you? I’m your son, too.” In both episodes, the young children believe that a biological connection makes them deserving of love and care, leaving them vulnerable to self-destructive decision-making. Longing for love from her biological father leads Cate to allow herself to be manipulated into a sexual connection with a much older man, and Arturo attacks his half-brother because he is unable to control his jealous rage. Cate had a loving, non-biological father, and Arturo had a loving mother. Yet, both children thirsted for love based on genetic kinship instead of valuing their existing social bonds. Ultimately, this worldview proves disastrous for both children, who cannot make sense of their confused biological relationships.

### Maternity

Although less common than a disruption in paternity, five of our surveyed episodes contained a question of biological maternity (S6.E1; S13.E4; S14.E7; S17.E8; S7.E11). Three of these episodes center on custody battles between a genetic relative and a social relative. Custody battles involving genetics in these episodes revolve around conflicting claims about the priority of biology vs. existing bonds forged by love and care. In all three, the child ends the episode in the custody of the original social relative, a rejection of biological primacy in determining parenthood. However, the journey to this resolution is fraught with conflicting emotions, and the detectives display sympathy for both parties. In “Birthright” (S6.E1), a girl in elementary school named Patty Branson is kidnapped by Michele Osborne, a widowed woman who claims Patty is her biological daughter. The detectives discover that Michele’s eggs were implanted in Sarah Branson during in vitro fertilization without the knowledge or consent of either woman. The resulting custody battle raises many questions about motherhood, social, legal, and genetic.

Sarah Branson: “I know you lost your daughter, but can’t you see what you’re doing to Patty?”

Michele Osborne: “She’s my flesh and blood.

Sarah Branson: “But she came from inside me. She kicked in my belly. She nursed from my breast.”

Michele Osborne: “She has my genes. And my husband’s genes.”

Sarah Branson: “Because of a mistake!”

Michele Osborne: “It wasn’t a mistake. My daughter died. And now God has given me a second chance.”

The women cling to their contrasting ideas of motherhood and their different forms of connection to Patty, social and biological. The detectives debate who should claim the child while the court case against Michele Osborne for kidnapping continues.

For most of the episode, the two women who claim to be Patty’s mother are at an impasse. Both truly believe that Patty should be theirs to house and to hold. The deciding factor, ultimately, is Patty. She does not want to go with Michele, her “egg mommy.” The detectives, the main arbiters of morality in this episode, are divided over who should have the child.

Detective Benson calls the biological mother’s kidnapping attempt a “mistake” driven by a desire to claim “her little girl,” suggesting that the biological relationship does allow awarding custody of the child. However, the biological mother loses Benson’s support when she continues to harass the Bransons. Once the genetic relationship interferes with a socially constructed family unit, the biological argument loses power. Once again, a character is driven to criminality and the edge of insanity as a result of newly gained genetic knowledge. Yet, the true question of motherhood is never fully decided, and the search for the answer pits the detectives against one another. The conclusion leaves room for individual interpretations of a tense on-screen debate.

Under the guise of showing both sides and leaving the answer to the viewer, the episode paradoxically divides the social from the biological as clear-cut categories. The extreme reaction of the genetic parent seems to indicate an absolute distinction between the claims of the biological and the social. But the essentialist beliefs of the genetic parent are themselves a social context, albeit one the show paints as destructive.

A later episode, “Vanity’s Bonfire” (S14.E7), tackles a similar version of the question of nature vs. nurture. The conflict arises between Dia Nobile, the child’s biological mother who was manipulated into giving up her child for adoption, and Josie Leddy, the child’s adoptive mother, who unknowingly received fraudulent adoption papers. Initially, Detective Benson supports a biological view of motherhood, telling the police captain, “By the look in her eye, Dia may have a connection to this baby.” However, Dia follows the same route as the other characters who are depicted as overvaluing their genetic claim by committing criminal acts of harassment and kidnapping. As Dia becomes more desperately attached to an essentialist conception of genetic relations, Detective Benson’s sympathy wanes. Dia’s monomaniacal focus on genetic relations at the expense of other social factors is presented as a kind of insanity. More distressing still, this critique of genetic essentialism seems to be leveled particularly at mothers.

### Detective Benson

For much of Season 19, Detective Benson is forced to combat essentialist claims about the priority of genetic relatedness in order to maintain custody of her adopted 5-year-old son, Noah Benson. At the start of the season, Noah’s biological grandmother, Sheila Porter (Brooke Shields), learns about Noah’s existence and attempts to reclaim Noah, citing a technical error in the adoption procedures. When she fails to vacate the legal adoption, Sheila works to build a positive relationship with both Noah and Benson over several episodes (S19.E5; S19.E6; S19.E8). In this period, a merely biological connection evolves into a loving social connection as both women express gratitude for the other and are happy to have a larger, extended family. For several episodes, Noah’s grandmother respects Benson’s bond with the adopted child and appears in a positive light. However, Noah’s grandmother loses all credibility in “Gone Baby Gone” (S19.E9) when she kidnaps Noah, convinced that her biological claim should outweigh Benson’s legal, emotional, and social connection. When Detective Benson tracks down the fugitive grandmother, they have a dramatic confrontation at gunpoint. A complex legal dispute about custody becomes a life-or-death confrontation that places genetic essentialism in the spotlight.

Detective Benson: “I’m his mother, Sheila.”

Sheila Porter: “Why? Because a judge signed a piece of paper? That doesn’t make you

his mother.”

Detective Benson: “Actually, actually Sheila it does.”

Sheila Porter: “No, he is my blood. He is my family. He will never be a part of you the way he is a part of me.”

Detective Benson: “Blood isn’t the only thing that makes families, Sheila. Legally, Noah is my son.”

Sheila Porter: “The law says that I would have received notice. If you followed the law, we wouldn’t be here.”

Detective Benson: “Sheila, Sheila, I am the only mother that he knows.”

Both sides have a legal argument for their claim over Noah, but any previous reasoning becomes secondary to the facts of their current situation. Sheila is a kidnapper and a criminal. Benson is the show’s protagonist and a devoted mother. Detective Benson never doubts her claim to Noah despite Sheila’s “blood” relationship to him. However, part of why Noah’s kidnapping is so heartbreaking to Benson is because she had found a sense of family with Sheila. Benson’s rejection of Sheila’s genetic claim is a necessity for Noah’s safety, not merely a principled stance, and the resolution is tinged with disappointment. With the show’s star ultimately turning away from genetic essentialism—professionally and personally—*SVU* promotes a higher valuation of social factors over biological ones.

Since *Law and Order: SVU* is centered on solving cases, the show’s ethical messaging is based on the who, what, and why of crimes. In this respect, the show’s heightened dramatic world differs radically from ordinary reality, where only a small minority of custody cases lead to violence and where essentialist thinking is intertwined with social assumptions. But for a crime show, the most effective way to present a negative judgment of an attitude or belief is to associate it with criminality. Time and again in *SVU*, belief in genetic essentialism motivates criminal behavior—a melodramatic exaggeration that unmistakably signals the show’s moral perspective. The path to a resolution may be full of complex emotional and social logic, but the concluding gesture almost always reduces this complexity to an either/or choice. In the end, *SVU* casts genetic essentialism in a negative light and endorses the idea that family is a social bond independent of genetics.

## Conflicting attitudes toward genetic determinism in SVU

In this section, we analyze claims on the show that heritable genetic predispositions can determine a person’s behavior. The two main manifestations of such genetic determinism in *SVU* involve debates about one’s responsibility for one’s actions and hopes that a child’s life course will be shaped by its parents’ DNA. First, we examine how *SVU* portrays the culpability of an individual for criminal acts when their genetics point to a predisposition to deviant behavior.

Next, we explore moments when the show predicts certain behaviors based on a person’s biology. In all these episodes, genetic information is discussed and contextualized by each party involved. The resulting debate and the testimony of fictional scientists during courtroom scenes create a multi-sided view of the issue of genetic determinism but only by artificially segregating the legal/scientific sphere from the personal/social sphere, as though each was not implicated in the other.

### Genetic predispositions for violence

Genetic predisposition to violence was central to three courtroom battles—two as a legal defense and one as the roadblock to crucial testimony (S3.E8, S5.E13, S18.E13). Although the criminals and their lawyers provide strong arguments in favor of genetic determinism, all three cases reject this position. Each of the three episodes feature an authority on genetics attempting to remove responsibility for a criminal action from the individual because of their genetic makeup, mimicking real-life criminal defenses based on neuroscience in the criminal justice system (Raine 2013). In “Genes” (S18.E13), a leader of a support group for the children of rapists tells the young people in the group, “Would you judge an alcoholic for taking a drink? He’s not responsible for his genetics. Neither are you.” The other two episodes rely on expert testimony by (fictional) scientists. “Hate” (S5.E13) features Dr. Emily Sopher (Linda Emond), cast in the role of a prominent neurobiologist. She advocates for the predictive power of genetics, saying, “Read the research on brain chemistry. Multiple studies, not just mine. Genotypes that cause low levels of monoamine oxidase A predict violence.” In the third episode, “Inheritance” (S3.E8), another fictional doctor testifies, “There is no violence gene per se. The violent behavior can be linked to the expression of several abnormal genes…. A defective DRD2 A-1 allele may lead to pleasure-seeking or addictive behavior as well as violence.” The doctor goes on to compare inheriting violent tendencies to inheriting cystic fibrosis, concluding that neither was chosen by the affected parties. All three defense witnesses assume that criminal behavior can be inherited from prior generations, asserting that the child’s sins are really those of the parent. The scientists and the counselor persuade some of the background characters in the show, but the detectives are never convinced and project themselves as the voices of reason who insist that we are in control of our own actions.

*SVU* allows a strong case to be made for the view that one’s DNA can lead one to commit a crime. However, the arguments fail to win the day in court. Here is the defense lawyer in “Inheritance” making a passionate plea for her client’s innocence based on his genetic predisposition to violence:

“Complex scientific evidence aside, this case is about the choices we make and the choices that are made for us. Did Darrell Guan choose for his father to rape his mother? To be brought up in a community, in a family that every single day berated, belittled, and stigmatized him? Did he choose to have a genetic defect? No. And now we’re supposed to tell him, ‘Well, too bad you should have known better.’ Darrell was born and lived in hell. His grandmother called him the devil. Well, of course, he was. He was engineered by nature and by nurture to be exactly what he turned out to be. And that is not his fault.”

For a moment, the jury appears convinced. However, ADA Casey Novak presents a closing argument that counters Darrell Guan’s lawyer and wins the case.

“Yes, Darrell Guan had a difficult life. He was raised knowing the only reason he existed was because his mother was violently raped. One would understand why he wouldn’t grow up to be a well-adjusted adult. But that’s not an excuse for murder. In a civilized society, we have to take responsibility for our actions. If we start to justify all antisocial behavior under the pretense it isn’t our fault, then the entire structure of the law is meaningless. In the end, it comes down to what it is to be a human being. We’re not just a product of our genetic programming. Nor are we solely molded and motivated by our childhood experiences. The defense would have you believe that Darrell Guan’s life was beyond his control. But the truth is, he was in complete control at the time he committed his crimes. He specifically chose his victims not because of some biological imperative but because of opportunity. He knew what he was doing was wrong and he did everything he could to get away with it. Darrell Guan chose the course of his life. Make him take responsibility for it.”

Neither lawyer disputes the facts of the case. Instead, they argue about how one should weigh the significance of a potential genetic predisposition toward violence. They both agree that individuals do not have a choice about their genetic makeup, but they differ over whether individuals have a choice about their actions. The jury finds Darrell Guan guilty, a formal rejection of the idea that his genes made him do it. *SVU*’s messaging is clear. The ADA offers a nuanced account of nature vs. nurture. Still, with the guilty verdict, the show affirms that people make choices and must take responsibility for them, regardless of genetics.

In “Hate,” the criminal’s lawyer claims his client, who engaged in a hate crime against Muslims, had no control over his bigotry because he “is biologically and genetically predisposed to hatred and violence.” The detectives are disgusted by the lawyer’s approach, making the heredity defense seem like a sly trick instead of a reasonable possibility. During the trial, the jury begins to believe that the criminal’s hatred of Muslims was encoded in his genes. However, the episode ends with the revelation of a motive for homicide that was more immediate: anger that his father had an Islamic woman as a mistress. Once the motivation for the suspect’s violent behavior is revealed, even his lawyer gives up on a genetic defense. Again, the suspect is shown to be in control of his actions instead of being led by a biological imperative.

In the third episode, “Genes,” Detective Benson herself struggles with the genetic implications of her father’s violence against women. Benson shares her concerns with a therapist who staunchly rejects the genetic determinism creeping into Benson’s view of herself.

Therapist: “Did you turn into your father?”

Detective Benson: “Well, I didn’t turn into a rapist if that’s what you mean. But I think about him all the time, and I think about what he did and the choices that he made. I think about it a lot. And I’ve been dealing with this for quite a long time, even before I met you. And no matter what I do, I still worry that… somehow his… darkness will overpower my life and my choices, because… I’ve been angry. I’ve been… violent.”

Therapist: “I know. And I know how much that frightened you, but I don’t believe that’s who you really are. My opinion: your strength, your moral compass, your belief systems… are more powerful than your father’s DNA. But there is no such thing as a rape gene, Olivia. Whether you’re male or female, there just isn’t.”

The therapist points Benson back to the values that guide her actions instead of a disheartening belief in genetic programming. But this conclusion ignores how nature and nurture are intertwined. The strength and moral compass that allowed Benson to resist her violent tendencies did not stem just from some vague and undefined “character”; they also came from all the biological and social advantages that enabled her to grow into the person she is, from her health and physical hardiness, her innate intelligence, and her upbringing. As a result of the therapist’s simplification, Benson overcomes her doubts and convinces an important witness to testify by assuring him, “There’s no such thing as a rape gene. There just isn’t. You have to trust me on that…. My father was a rapist. So, I know this one. I’ve been dealing with it for my whole life. But that doesn't make me bad. That doesn't make me evil.” The witness is convinced, and the resulting testimony successfully convicts the defendant. Once again, an episode is structured so that the idea of genetic determinism is an obstacle that the detectives must overcome to obtain justice. The perpetrator of a crime is made to take responsibility for his actions, denying that genetics are a script that one is doomed to follow.

Although their conclusions are decisive, the three episodes present reasoned cases along the way for the existence of a genetic predisposition toward violence. The shows lend an air of plausibility to the idea of genetic determinants of behavior for the audience to consider. By portraying both sides of the case, *SVU* makes its critique of genetic determinism more nuanced. Yet the final answer is clear. In the legal world presented in *SVU*, the defense “My genes made me do it” is rejected.

### Detective Stabler

In the episode concerning Stabler’s daughter and mental illness, the tone shifts. The other episodes were staunchly opposed to a genetics-based defense over fear that a legal victory for genetic determinist principles would mean “no one is truly responsible for their own actions” (S3.E8). However, Detectives Stabler and Benson become comfortable with a defense based on genetics when Stabler’s daughter is caught breaking into someone’s home during a manic episode resulting from her undiagnosed bipolar disorder (S10.E3). Haunted by his traumatic experiences with his mother, Stabler is determined to protect his daughter but cannot convince her to accept needed psychiatric treatment. Like his daughter, Stabler’s mother has never fully admitted that she suffers from bipolar disorder. The episode follows Stabler as he attempts to convince his mother to acknowledge her bipolar disorder so that his daughter’s lawyer can argue that a genetic predisposition to bipolar disease means that she should be sent to a psychiatric facility instead of jail. Ultimately, Stabler’s mother agrees to meet her granddaughter and tell her about a time she crashed a car during a manic episode. The grandmother’s analysis of the situation mirrors while inverting the conversations around culpability found in episodes concerning genetic predispositions to violence. Here, the daughter must accept responsibility for her actions, despite their origin in a heritable bipolar condition, in order to be persuaded to seek treatment for her disease. The grandmother tells her: “See, we all make mistakes, Katie. There’s no shame in that. But you do have to take responsibility for your actions.” When the daughter asks, “Do you think I’m crazy?” the grandmother replies: “I think you’re different. Like me. I’ve lived the life I wanted to live, and I’ve paid a terrible price.” For the daughter, taking the appropriate level of responsibility for her actions means accepting her genetics-based bipolar disorder and getting treatment.

Across the four episodes we have examined, the theme of responsibility recurs as an essential part of managing genetic predispositions. Although genetics is granted a certain influence over behavior, arguments that remove all responsibility for our actions are always rejected. *SVU* portrays genetic predispositions as relevant only when the person is working through their difficulties. When a sly defense attorney wants to set free all suspects who have inherited a predisposition for violence, the detectives reject this denial of personal choice.

Similarly, when managing a genetic predisposition to mental illness, one must first acknowledge that one has a choice to seek to understand one’s condition. Stabler’s daughter benefits from a version of the genetic defense. Still, in caring for herself by accepting her diagnosis and going into therapy, the daughter is simultaneously rejecting the idea that genetics determines how she must act.

In the episodes featuring genetic determinism, *SVU* presents characters of different backgrounds who believe strongly in the power of genetics to govern behavior and personal development. These characters—often full of confidence and armed with plausible-sounding scientific evidence—promote genetics as a tool that can be used to shape a child’s future. Yet the show ultimately rejects these arguments. Through the judicial system or the detective’s reaction, *SVU* conveys its dissent from this kind of determinist thinking. To the detectives and the ADAs, valuing genetics too highly could undermine the entire structure of the legal system, which is based upon individuals taking responsibility for their actions and paying their debt to society.

While the pro-and-con structure of legal argumentation leaves room for viewers to consider the question of genetic determinism for themselves, the ultimate rejection of genetic determinism relies on personal and social beliefs about character and responsibility.

## Conclusion

If *Law and Order: Special Victims Unit* seems to assert that “DNA doesn’t lie,” the truth DNA tells on this popular TV show is complex and needs interpretation. In the episodes we examined, the importance of genetics was consistently shown to be complicated by other social factors, so difficult ethical and legal questions of kinship, custody, and behavior in non-forensic contexts were rarely if ever, settled by a simple reliance on genetic evidence.

Essentialist and deterministic perspectives on the impact of genes on identity, family, and behavior were aired in multiple contexts beyond forensics, but counterarguments that emphasized the role of loving relationships, a history of care, existing family arrangements, and other entitlements generally won out in the end.

Although *SVU* rejects genetic essentialism and determinism by portraying social factors as trumping biological factors, it does so by overemphasizing the difference between nature and nurture. The series gives viewers the latitude to interpret the importance of DNA in shaping character and behavior for themselves, but the necessity of resolving the case at the end of the episode pushes the show to make one win out over the other. In doing so, it separates nature and nurture instead of building a complex, nuanced understanding of identity and family that acknowledges the power of both the social and the biological. In portraying the overlapping and interrelated factors as independent, *SVU* perpetuates an outdated distinction between nature and nurture.

Science is a part of culture, and the way it is portrayed in popular media affects the way viewers understand it and its social implications. As the most popular crime drama in television history, *SVU’*s role in shaping public perceptions of genetics is significant. In and outside the courtroom, the series dispels one misconception by reinstating another. Even when *SVU* undermines genetic essentialism and determinism, it relies on a narrative of conflict between nature and nurture to do so.

## Data Availability

All data included in the Appendix of the article.

## Appendix

Episodes examined.

**Table:**

Season and episode #	Title of episode	Date aired	Genetic essentialist themes	Genetic determinist themes
S1.E17	“Misleader”	March 31, 2000	X	
S3.E8	“Inheritance”	November 16, 2001		x
S3.E22	“Competence”	May 10, 2002		
S4.E8	“Waste”	November 15, 2002	X	
S4.E14	“Mercy”	January 31, 2003		
S4.E18	“Desperate”	March 14, 2003	X	
S4.E24	“Perfect”	May 9, 2003		
S5.E5	“Serendipity”	October 21, 2003		
S5.E13	“Hate”	January 13, 2004		x
S6.E1	“Birthright”	September 21, 2004	X	
S6.E12	“Identity”	January 18, 2005		
S7.E2	“Design”	September 27, 2005	X	x
S7.E11	“Alien”	December 6, 2005	X	
S7.E14	“Taboo”	January 17, 2006	X	
S8.E2	“Clock”	September 26, 2006	X	
S8.E5	“Confrontation”	October 17, 2006	X	x
S8.E16	“Philadelphia”	February 27, 2007	X	x
S8.E19	“Florida”	May 1, 2007	X	
S8.E22	“Screwed”	May 22, 2007	X	
S9.E9	“Paternity”	November 27, 2007	X	
S10.E3	“Swing”	October 14, 2008		x
S12.E2	“Bullseye”	September 22, 2010	X	
S13.E3	“Blood Brothers”	October 5, 2011	X	
S13.E4	“Double Strands”	October 12, 2011	X	
S13.E16	“Child’s Welfare”	February 29, 2012	X	
S13.E20	“Father Dearest”	May 2, 2012	X	
S14.E7	“Vanity’s Bonfire”	November 14, 2012	X	
S16.E15	“Undercover Mother”	February 18, 2015	X	
S16.E23	“Surrendering Noah”	May 20, 2015	X	
S17.E8	“Melancholy Pursuit”	November 11, 2015	X	
S18.E13	“Genes”	March 22, 2017		x
S19.E3	“Contrapasso”	October 11, 2017	X	
S19.E5	“Complicated”	October 25, 2017	X	
S19.E6	“Unintended Consequences”	November 1, 2017	X	
S19.E8	“Intent”	December 6, 2017	X	
S19.E9	“Gone Baby Gone”	January 3, 2018	X	
S20.E5	“Accredo”	October 18, 2018	X	
S20.E20	“The Good Girl”	April 11, 2019	X	
